# Efficacy of isoproterenol for treating amlodipine overdose resulting in bradycardia

**DOI:** 10.1002/ams2.284

**Published:** 2017-05-26

**Authors:** Takeshi Ebihara, Masanori Morita, Masahiro Kawada, Koji Amano, Fumitaka Kato, Yasuki Nakata

**Affiliations:** ^1^ Department of Critical Care Medical Center Sakai City Medical Center Osaka Japan; ^2^ Department of Emergency and Critical Care Medicine Saiseikai Shiga Hospital Shiga Japan; ^3^ Present address: Sakai City Medical Center on April 1, 2017

**Keywords:** amlodipine, bradycardia, isoproterenol, overdose

## Abstract

**Case:**

Amlodipine predominantly affects vascular smooth muscle cells. Amlodipine overdose usually presents with vasodilatory shock, accompanied by reflex tachycardia rather than bradycardia.

An 81‐year‐old woman presented with impaired consciousness 8 h after ingesting 50 5‐mg amlodipine tablets with suicidal intent. On admission, her blood pressure was 50/40 mmHg and her heart rate was 45 b.p.m. Serum amlodipine level was extremely high (474.4 ng/mL), causing refractory bradycardia. She remained hypotensive despite fluid resuscitation, and therefore was administered dopamine and norepinephrine. She was also administered glucagon and calcium gluconate, and underwent high‐dose insulin euglycemic therapy.

**Outcome:**

Although her blood pressure improved, bradycardia progressively worsened and isoproterenol infusion was initiated, which resulted in an improvement in her heart rate. The patient discharged on day 14 without any complications.

**Conclusion:**

Isoproterenol is effective for treating bradycardia after amlodipine overdose.

## Introduction

Amlodipine is a dihydropyridine calcium‐channel blocker (CCB) that predominantly affects vascular smooth muscle cells, and causes marked peripheral vasodilation and hypotension. Amlodipine overdose usually presents with vasodilatory shock, accompanied by reflex tachycardia rather than bradycardia. Here, we report a case of amlodipine overdose with refractory bradycardia effectively treated with isoproterenol infusion.The protocol for this research project was approved by a suitably constituted Ethics Committee of the institution and it conforms to the provisions of the Declaration of Helsinki. The Ethics Committee permitted publication of this clinical case without registration. The patient provided her informed consent for her anonymized data to be used in this report.

## Case

An 81‐YEAR‐OLD woman with a history of hypertension treated with only amlodipine was admitted to our emergency department with impaired consciousness 8 h after ingesting 50 5‐mg amlodipine tablets with suicidal intent. On admission to the emergency department, blood pressure (BP) of 50/40 mmHg and heart rate (HR) 45 b.p.m. were recorded. Her Glasgow Coma Scale score was 13. The results of routine laboratory tests on admission were as follows: total leukocyte count 10,140/μL, serum creatinine 1.37 mg/dL, blood urea nitrogen 22.2 mg/dL, serum ionized calcium 1.14 mmol/L, and lactate 5.5 mmol/L. A 12‐lead electrocardiogram revealed no abnormalities. Comprehensive toxicological analysis of the urine was negative.

**Figure 1:**
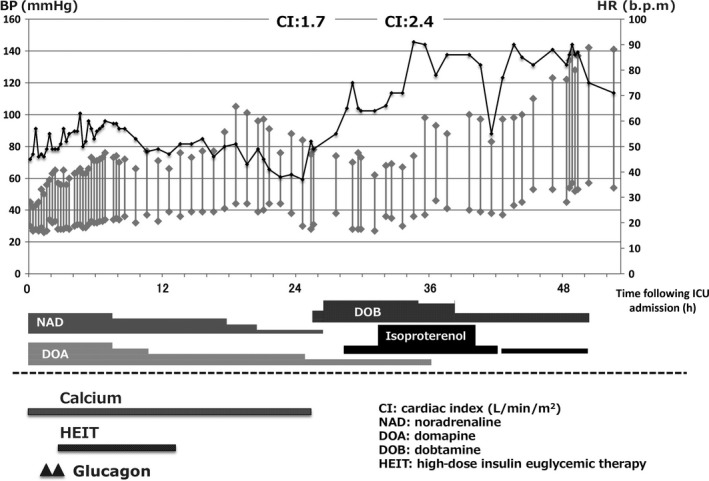
Clinical course of an 81‐year‐old woman with amlodipine overdose with hypotension and refractory bradycardia treated with calcium, glucagon, and high‐dose insulin euglycemic therapy (HEIT) with various vasopressor agents and isoproterenol. Isoproterenol is effective for improving bradycardia. BP, blood pressure; HR, heart rate; ICU, intensive care unit.

**Figure 2:**
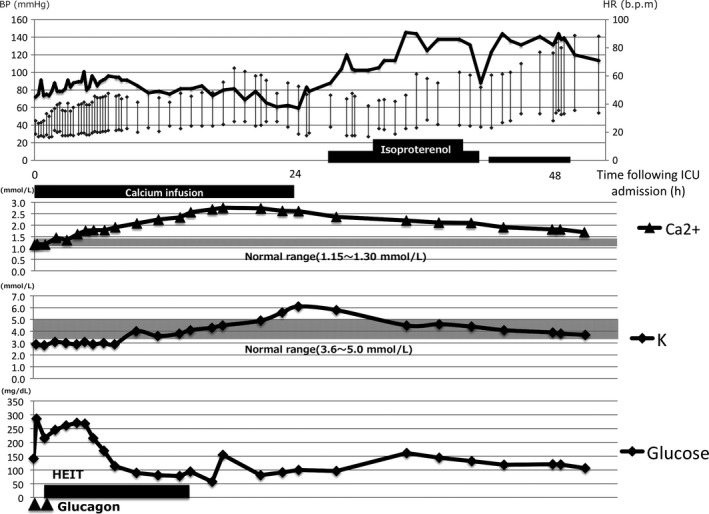
Relationship between hemodynamic parameters and serum ionized calcium level, potassium level, and glucose level in an 81‐year‐old woman with amlodipine overdose with hypotension and refractory bradycardia. The serum calcium level was up to two times the upper limit of the reference range following continuous calcium infusion. Isoproterenol is effective for improving bradycardia. High‐dose insulin euglycemic therapy (HEIT) was discontinued because the patient developed hypoglycemia 13 h after admission to the intensive care unit (ICU). BP, blood pressure; HR, heart rate.

Her medical history revealed only hypertension, treated with amlodipine, without a β‐blocker or angiotensin‐converting enzyme inhibitor. She was not diagnosed with depression before admission. The patient was diagnosed with an amlodipine overdose. She was intubated for airway protection and given fluid boluses. Intravenous vasopressor therapy was initiated (dopamine, 10 μg/kg/min; noradrenaline, 0.2 μg/kg/min). Atropine showed no effect on HR. She was hospitalized in the intensive care unit (ICU). The clinical course of the patient is shown in Figure 1. On admission to the ICU, her BP was 50/35 mmHg with an HR of 40 b.p.m. She remained in a hypotensive state despite high‐dose vasopressor therapy. A calcium gluconate (8.5%) infusion was initiated at a rate of 0.7 mL/kg/h, and her serum ionized calcium level was maintained at up to two times the upper limit of the reference range. On admission to the hospital, hyperglycemia was not shown. In this case, glucagon was given as a 100‐μg/kg bolus followed by further doses at a rate of 100 μg/kg/h. In addition, high‐dose insulin euglycemic therapy (HEIT) was initiated by an insulin infusion at a rate of 15 units/h with 10% dextrose. Despite these interventions, the patient's hemodynamic parameters did not improve. High‐dose insulin euglycemic therapy was subsequently stopped because the patient developed hypoglycemia 13 h after admission to the ICU. As the patient's serum ionized calcium level increased, her BP improved (Fig. 2). The doses of noradrenaline and dopamine were decreased, but bradycardia continued to worsen. After 24 h in the ICU, the serum ionized calcium level peaked at 2.7 mmol/L. Blood pressure fell despite a consistently high serum ionized calcium level. A dobutamine infusion was started and was increased up to 6 μg/kg/min to improve cardiac output with little effect. Despite these interventions, the patient's HR remained at 30–50 b.p.m., therefore, an isoproterenol infusion was started 26 h after ICU admission to improve cardiac output by increasing the HR. Soon after the infusion was started, the patient's HR increased. After 48 h in the ICU, the patient's serum ionized calcium level decreased to 1.5 mmol/L, and her HR and BP remained stable. Isoproterenol was weaned and stopped on ICU day 3. The patient was extubated on the same day, and she was discharged on day 14 without any complications.

## Discussion

Calcium‐channel blockers are among the most widely used treatments for hypertension, angina pectoris, and congestive heart failure. The American Poison Control Centers reported that cardiovascular drugs have the fourth greatest rate of increase of serious outcomes.^1^ According to the National Poison Data System, CCBs were responsible for at least 17,720 exposures and 52 deaths in 2014 in the USA alone.^1^ Expert consensus recommendations for the management of CCB poisonings were published and stepwise management was recommended. However, unfortunately, there are no strongly recommended first‐line treatments for refractory patients and the level of evidence was very low.^2^ Therefore, a multimodal therapeutic approach is often used according to the situation.^3^, ^4^, ^5^

Calcium‐channel blockers are categorized into three classes according to differences in chemical structure: phenylalkylamines, benzothiazepines, and dihydropyridines. Due to pharmacological selectivity, dihydropyridine CCBs (e.g. amlodipine, nifedipine) primarily affect the calcium channels in the smooth muscle^6^ and, in cases of overdose, cause vasodilatory shock with reflex sinus tachycardia.^7^, ^8^ Phenylalkylamines such as verapamil and benzothiazepines such as diltiazem cause cardiogenic shock in the case of overdose. Amlodipine overdose with β‐adrenergic blockers or the other classes of CCBs do not present with reflex sinus tachycardia. In fatal cases, cardiac arrest can occur and extracorporeal life support is required to rescue the patient.^9^

In the present case, refractory bradycardia continued after admission to the hospital. The serum amlodipine level was 474.43 ng/mL at admission and 251.75 ng/mL 24 h later. These levels are extremely high compared to the maximum concentration produced by a single oral dose of amlodipine (5 mg) reported in a study of 16 healthy elderly people^10^ (3.0 ± 1.1 ng/mL) and the peak serum amlodipine level reported in a study of six elderly Japanese patients given amlodipine (5 mg) daily for 8 days (14.9 ± 2.2 ng/mL) (published only in Japanese)^11^. A high serum amlodipine level causes loss of pharmacological selectivity. This could explain why the patient in this report experienced bradycardia with severe hypotension rather than reflex sinus tachycardia, as may be expected.

**Figure 3:**
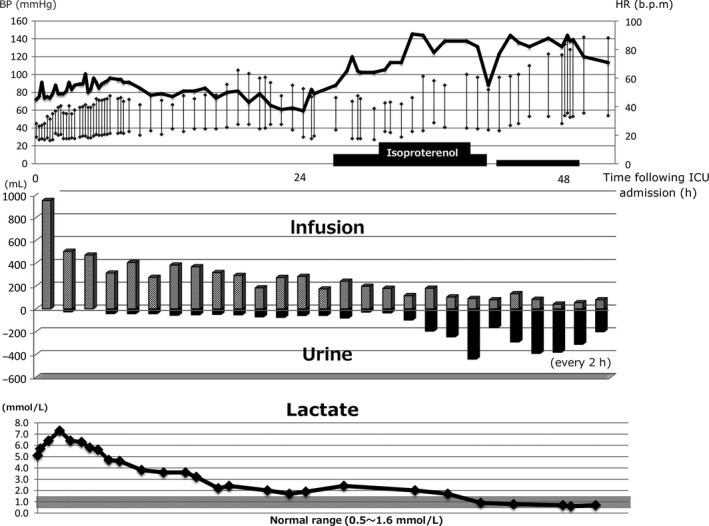
Relationship between hemodynamic parameters and in–out balance and serum lactate level in an 81‐year‐old woman with amlodipine overdose with hypotension and refractory bradycardia. The patient's condition began improving once her heart rate (HR) increased, and diuresis and normalization of the serum lactic acid level were also observed. BP, blood pressure; ICU, intensive care unit.

In the present case, the serum calcium level was up to two times the upper limit of the reference range following continuous calcium infusion. Hypotension improved, but oliguria and lactic acidosis continued. Dobutamine infusion was not enough to improve the patient's hemodynamic condition, thus, isoproterenol infusion was started to improve cardiac output by increasing the HR. The patient's condition began improving once the HR increased, and diuresis and normalization of the serum lactic acid level were also observed (Fig. 3). A FlowTrack (Edwards Lifesciences, Irvine, CA, USA) was used to monitor the hemodynamic parameters, and the cardiac output index increased from 1.7 to 2.4 L/min/m^2^ after starting the isoproterenol infusion. Isoproterenol is a non‐selective β‐adrenoreceptor to treat bradycardia associated with diltiazem and verapamil overdoses, which cause cardiotoxicity.^12^ Based on the current report,^13^ isoproterenol was used at 5–25 μg/min (maximal infusion rate is 60 μg/min). In our case, we started isoproterenol at 12 μg/min and increased up to 30 μg/min. Because bradycardia is not often present in amlodipine overdose cases, there are no reports that used isoproterenol for amlodipine overdose. In our case, isoproterenol prevented the use of a pacemaker or extracorporeal life support. Isoproterenol may be effective for refractory bradycardia caused by extremely high serum amlodipine.

The use of calcium to treat CCB poisoning is physiologically reasonable and clinically indicated, but the response to calcium is also inadequate.^3^, ^4^, ^5^ A systematic review^4^ reported that adverse effects of therapeutic calcium infusion are rare following CCB poisoning. In the present case, the patient's BP improved in proportion to rising serum calcium levels, but her HR decreased. This finding was surprising, as calcium infusion has been reported to improve BP, but no reports to date have documented that calcium has an effect on HR.^4^ Mild hyperkalemia (*K* = 6.0 mmol/L) due to acute renal failure was also present. It is unclear whether the progressively worsening bradycardia reported in this case was related to the adverse effects of artificial hypercalcemia due to continuous calcium infusion or hyperkalemia. However, acute hypercalcemia causes bradycardia,^14^ and artificial hypercalcemia could have contributed to worsening bradycardia.

In conclusion, isoproterenol infusion is a promising treatment for hypotension with bradycardia caused by amlodipine overdose.

## References

[1] Mowry JB , Apyker DA , Brooks DE . ‘2014 annual report of the american association of poison control centers’ national poison data system (NPDS): 32nd annual report. Clin. Toxicol. 2015; 53: 962–1147. 10.3109/15563650.2015.1102927 26624241

[2] Maude St , Kurt A , Frank LC . Experts consensus recommendations for the management of calcium channel blocker poisoning in adults. Crit. Care Med. 2017; 45: e306–15. 27749343 10.1097/CCM.0000000000002087 PMC5312725

[3] Shepherd G . Treatment of poisoning caused by beta‐adrenergic and calcium‐channel blockers. Am. J. Health Syst. Pharm. 2006; 63: 1828–35. 16990629 10.2146/ajhp060041

[4] Graudins A , Lee HM , Druda D . Calcium channel antagonist and beta‐blocker overdose: antidotes and adjunct therapies. Br. J. Clin. Pharmacol. 2016; 81: 453–61. 26344579 10.1111/bcp.12763 PMC4767195

[5] St‐Onge M , Dubé PA , Gosselin S . Treatment for calcium channel blocker poisoning: a systematic review. Clin. Toxicol. 2014; 52: 926–44. 10.3109/15563650.2014.965827 PMC4245158 25283255

[6] DeWitt CR , Waksman JC . Pharmacology, pathophysiology and management of calcium channel blocker and beta‐blocker toxicity. Toxicol. Rev. 2004; 23: 223–38. 15898828 10.2165/00139709-200423040-00003

[7] Patel T , Tietze D , Mehta AN . Amlodipine overdose. Proc. (Bayl. Univ. Med. Cent.) 2013; 26: 410–1. 24082424 10.1080/08998280.2013.11929022 PMC3777093

[8] Upreti V , Ratheesh VR , Dhull P . Shock due to amlodipine overdose. Indian J. Crit. Care Med. 2013; 17: 375–7. 24501491 10.4103/0972-5229.123452 PMC3902574

[9] Maskell KF , Ferguson NM , Bain J . Survival after cardiac arrest: ECMO rescue therapy after amlodipine and metoprolol overdose. Cardiovasc. Toxicol. 2017; 17: 223–5. 26913719 10.1007/s12012-016-9362-2

[10] Elliott HL , Meredith PA , Faulker JK . A comparison of the disposition of single oral doses of amlodipine in young and elderly subjects. J. Cardiovasc. Pharmacol. 1988; 12: 64–6. 10.1097/00005344-198812007-00014 2467132

[11] Kuwajima I , Suzuki Y , Kumamoto K . Study of pharmacokinetics of amlodiphine in essential hypertension of elderly people. Geriat.Med. 1991; 29: 899–902.

[12] Ramoska EA , Spiller HA , Winter M . A one‐year evaluation of calcium channel blocker overdoses: toxicity and treatment. Ann. Emerg. Med. 1993; 22: 196–200. 8427431 10.1016/s0196-0644(05)80202-1

[13] Michael L , Steven CC , Angela P . Critical care management of verapamil and diltiazem overdose with a focus on vasopressors: a 25‐year experience at a single center. Ann. Emerg. Med. 2013; 62: 252–8. 23642908 10.1016/j.annemergmed.2013.03.018

[14] Badertscher E , Wamica JW , Emst DS . Acute hypercalcemia and severe bradycardia in a patient with breast cancer. CMAJ 1993; 148: 1506–8. 8477369 PMC1491861

